# Impact of climate change on public health in Brazil

**DOI:** 10.1002/puh2.62

**Published:** 2023-01-28

**Authors:** Mariana Abou Mourad Ferreira, Yuri Luiz Reis Leite, Crispim Cerutti Junior, Creuza Rachel Vicente

**Affiliations:** ^1^ Programa de Pós‐Graduação em Doenças Infecciosas, Centro de Ciências da Saúde Universidade Federal do Espírito Santo (UFES) Vitória Espírito Santo Brazil; ^2^ Programa de Pós‐Graduação em Ciências Biológicas, Centro de Ciências Humanas e Naturais Universidade Federal do Espírito Santo (UFES) Vitória Espírito Santo Brazil

**Keywords:** Brazil, climate change, deforestation, global health, temperature

## Abstract

Brazil is South America's largest country and economy, represented mainly by agricultural commodities. Its vast rainforest and biodiversity are at constant risk from human actions that are seen by scientists contributing to climate change. This article dissects how Brazil influences and is directly and indirectly affected by climate change and possible strategies to control the current situation. Climate change impacts Brazilian public health in multi‐scenarios and is influenced by socioeconomical and geopolitical aspects, such as urbanization, access to sanitation and sewage, precipitation intensity and frequency, and public health policies. Therefore, surveillance and control measures, alongside socioeconomic policies, must be orchestrated to minimize human actions that impact climate change.

## Graphical abstract

Brazil's rainforest and biodiversity are at constant risk from human actions contributing to climate change.

Surveillance and control measures, alongside socioeconomic policies, must be orchestrated to minimize the impact of climate change.

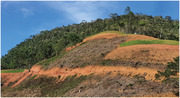

## INTRODUCTION

The shifts in temperature and rainfall that characterize climate change impose health consequences, especially on the vulnerable population. Brazil is susceptible to climate change impacts, and with over a third of worldwide tropical rainforests, it plays an essential role in responding to this global threat [1]. On the other hand, as one of the world's largest exporters of agricultural commodities, the country is responsible for the highest atmospheric greenhouse gas emissions due to land use changes, with high deforestation rates [2]. Per se, deforestation is a health threat, with habitat fragmentation contributing to human exposure to reservoirs and vectors of zoonotic pathogens and, consequently, to the emergence of infectious diseases [3]. However, Brazil's six major continental biomes (Amazon, Cerrado, Atlantic Forest, Caatinga, Pampas, and Pantanal) have different vulnerability levels to climate changes, including those caused by deforestation, and actions should therefore consider the particularities of each region [4].

## IMPACT OF CLIMATE CHANGE ON HEALTH IN BRAZIL

Brazil is a hotspot of emerging infectious diseases due to its location in a tropical region, rich biodiversity, and extensive land use [5]. All areas of the country present a risk for emerging zoonosis, including viral hemorrhagic fevers. In addition, pathogens introduced in the Brazilian territory caused large outbreaks, leading to hospitalizations and deaths, such as dengue and yellow fever [6]. Besides, the Amazon rainforest probably hosts many potential pathogens that have not been discovered and studied yet. Climate change may exacerbate this risk as infectious disease outbreaks in Brazil are frequently linked with climate change events, such as El Niño, La Niña, heat waves, droughts, floods, increased temperature, and higher rainfall [7]. Climate change may also contribute to other current health problems, such as emergencies, disasters, impacts on mental health, and increasing chronic diseases, especially linked with unplanned urbanization, which is now 86% in Brazil, with predictions to rise to 92% in 2050 [1].

Fires play a substantial role in climate change, especially in releasing harmful gases into the atmosphere. Those pollutants may cause serious health problems like cancer, DNA damage, and respiratory diseases. The Amazon rainforest and Pantanal wetlands faced one of the worst fire seasons in the past few years. Even when anthropogenic factors might not have directly caused fires, the government's poor management and underestimation of its magnitude worsened the situation. On top of that, fires are responsible for pathogen spillover and habitat loss leading to human and animal migration, causing the urbanization of green areas and the spread of vectors, including mosquitoes [8].

Dengue, Zika, and chikungunya are diseases transmitted by mosquitoes, primarily *Aedes aegypti*, which bring burden to Brazil, especially in urban areas where this species is very well adapted. A better understanding of the ecological systems of mosquito‐borne diseases and their environment is crucial to anticipate the emergence of vector‐borne epidemics [9]. Climate change will impose additional challenges to controlling these diseases due to the influence of high temperatures in the life cycle and behavior of the mosquitoes. Besides, rainfall changes may contribute to breeding sites, especially in places with poor sanitation [10]. Therefore, the recrudescence of the epidemiological scenario for these diseases is expected [7], including their territorial expansion in the country. Similarly, other vector‐borne diseases, such as malaria and yellow fever, may increase their distribution. Although these are typically prevalent in wild regions of Brazil, they may go through urbanization in the future due to climate and environmental changes.

Inadequate sanitation and unplanned urban occupation also lead to successive disasters linked with adverse climate events, such as floods and landslides. For example, between 2000 and 2018, Brazil registered 65 floods, representing 71% of the disasters recorded, with 2435 deaths, meaning 88% of the fatalities due to disasters [11]. Another study, based on the Brazilian Atlas of Natural Disasters, reports that flash floods were the disaster that led to the most number of deaths between 1991 and 2012, followed by landslides and inundation [12]. Beyond the deaths and social consequences, these events may be followed by outbreaks of water‐borne diseases, such as leptospirosis, which is predicted to increase in Brazil with climate change [13]. In addition, floods raise risks for the reemergence of cholera, of which the last outbreak in Brazil occurred in 1991 and the last autochthonous case in 2005 [14].

The temperature‐increased projections show warning heat stress conditions, with areas presenting high and extreme risks to human health, especially in the Northern and Center‐Western Brazilian regions. Ischemic health disease is the leading cause of death in Brazil and may worsen because heat waves will impact mortality by cardiovascular [15]. In addition, respiratory disease deaths may increase due to heat waves. Regarding cardiovascular diseases, the fraction of deaths attributed to heat stress, with a global warming of +4°C, is predicted to be 40% in the Northern, 35% in the Northeastern, and 25% in Center‐West capitals [16]. Heat waves have already been responsible for increased mortality and preterm births during record‐breaking droughts in southeastern Brazil between 2013 and 2015 [17]. In Brazil, heat exposure is associated with an increase in all‐cause hospitalizations and those due to ischemic heart disease, asthma, pneumonia, renal diseases, mental health conditions, and neoplasms, impacting the demand for medical and surgical care [18].

Beyond the consequences of global warming for the Brazilian population, it is well known that social inequities determine disparities in how communities experience climate change risks [19]. Earlier quantitative assessment of the vulnerability of the Brazilian population to the health impacts of climate change found that the socioeconomic vulnerability index had the most substantial influence among all indicators [20]. In addition, the Northeastern region was the most vulnerable to the health impacts of a changing climate [20]. Generally, a stronger association is found in less developed cities, which may exacerbate their existing inequalities [18]. Along with this, food production and water availability may be affected, increasing famine and imposing further health and social consequences. Therefore, raising the visibility of vulnerable groups, such as the indigenous population, is essential in health policy and actions to address the consequences of climate change in this country. Thus, Brazil included vulnerability and adaptation assessments to identify weaknesses in the health systems that should protect the most vulnerable populations from climate change risks [21].

Biodiversity conservation is vital for ecosystems and provides health benefits. In the Amazon, for example, malaria, acute respiratory infection, and diarrhea occurrence are significantly and negatively correlated with the area under strict environmental protection. Conservation scenarios suggest that the incidence of these diseases would be reduced by expanding strictly protected areas, and malaria could be further reduced by restricting roads and mining [22].

Although usually neglected, mental health is heavily impacted by changes in climate. The most common psychological manifestations are anxiety, stress, depression, post‐traumatic stress disorder, and suicide [23]. Those can arise after acute events, such as hurricanes and wildfires, or long‐term episodes, like higher temperatures and rising sea levels. In Brazil, the recent environmental disasters eliciting geophysical changes, like the 2015 Mariana and the 2019 Brumadinho tailing dam catastrophic failures, led to toxic particles reaching the river and water sources, in addition to almost 300 direct deaths. In addition, these events affected the local population, causing job losses, migration, and food insecurity, directly linked with mental health consequences.

## CURRENT POLICIES AND CHALLENGES

Many obstacles must be overcome to control and prevent climate emergencies effectively, as they are essential drivers of emerging infectious diseases. Notably, the World Health Organization (WHO) 2021 report enumerated the following list of responses that a country should do to prepare for those emergencies: (i) document evidence to support actions; (ii) develop national plans and policies, with intersectoral collaboration among stakeholders; (iii) prevent risks with surveillance and warning systems; (iv) create and manage funds to face ongoing, and future impacts of climate change; and (v) point out co‐benefits of climate change mitigation to raise incentives [21].

Along with that, the National Adaptation Plan (NAP), established in 2010, advocates a strong response to climate change, especially among vulnerable countries, including Brazil. The WHO's recent review of NAPs points to the importance of a targeted approach regarding different contexts and incidents. According to it, all the 19 reviewed NAPs mention water‐borne and vector‐borne diseases as climate‐sensitive health risks, though not all have developed an action plan to tackle those risks [24].

The Brazilian Universal Health System (Sistema Único de Saúde—SUS) is a global example of its actions on minimizing inequalities through addressing, among all, the most vulnerable population. Regarding climate change, the Health Surveillance Department coordinates the main actions in a centralized, generic way [25]. Alternatively, strategies should also be performed in non‐health divisions, such as meteorological sectors, helping to manage risk by warning of future weather events. Another example is improving the sanitation infrastructure, which is constantly impacted by climate events like rainstorms leading to inundations.

In 2016, Brazil completed the national health and climate change plan. Nevertheless, its implementation is ongoing, with the absence of an operational multi‐stakeholder mechanism established by the ministry of health. Furthermore, no memorandum of understanding was agreed upon by the ministry of health and the sectors of agriculture, education, energy, social services, transportation, urban development and housing, water, sanitation, and hygiene, compromising the definition of specific roles and responsibilities in policies or programs dealing with health and climate change [21].

## CONCLUSION AND FUTURE PERSPECTIVES

The data published in this study suggest that public policies should be reinforced, interconnected with agricultural, demographic, and environmental information, and funded internationally, especially in a developing country such as Brazil, with a significant impact on—and vulnerable to—climate change. Indeed, all the response is based on what is assessed; therefore, implementing adequate tools will estimate impacts and track indicators. Strategies may focus on the uniqueness of each region, such as biomes, protected areas, social circumstances, and agricultural businesses. Some strategies could include (i) for agriculture, the cultivation of species varieties that require less water and warmer temperatures, with investment in agricultural engineering; (ii) for water resources, the reuse of water and the use of rainwater, with economic incentives; (iii) for infrastructure, the protection of natural barriers, the building of green spaces, and the use of sustainable materials, with economic incentives and local policies.

Also, actions toward technologies and innovative approaches should be reinforced and financed, such as renewable energy resulting in low carbon emissions. Thus, climate change actions may lead to multiple co‐benefits, such as cost cuttings and an overall health amelioration.

Despite presenting many impacts of climate change in Brazil, this topic is complex and indirectly connected to several other relevant aspects. Above all, we intended to focus on the most common problems in Brazil. Our limitations include not assessing the influences of the COVID‐19 pandemic on climate change due to the scarcity of studies and tools to assess its environmental impacts in Brazil. During the COVID‐19 pandemic, telemedicine possibly reduced carbon emissions due to lower transportation use. On the other hand, there was an extensive consumption of single‐use plastics in medical settings. Therefore, investments in developing more sustainable alternatives for materials for medical and surgical care may reduce the health‐care carbon footprint, contributing to the reach of the success of the resolution of the United Nations about the right to a clean, healthy, and sustainable environment.

## AUTHOR CONTRIBUTIONS

Mariana Abou Mourad Ferreira, Yuri Luiz Reis Leite, Crispim Cerutti Junior, and Creuza Rachel Vicente contributed to data curation, formal analysis, and writing.

## CONFLICTS OF INTEREST

M.A.M.F., Y.L.R.L., and C.C.J. declare no conflicts of interest. C.R.V. is an editorial board member of the journal. C.R.V. was excluded and blinded from all stages of the peer review of this manuscript. No writing assistance was utilized in the production of this manuscript.
